# Plasmonic Effect of Gold-Patchy Silica Nanoparticles on Green Light-Photopolymerizable Dental Resin

**DOI:** 10.3390/nano13182554

**Published:** 2023-09-13

**Authors:** Melinda Szalóki, István Csarnovics, Attila Bonyár, Ditta Ungor, Edit Csapó, András Sápi, Csaba Hegedűs

**Affiliations:** 1 Department of Biomaterials and Prosthetic Dentistry, Faculty of Dentistry, University of Debrecen, H-4032 Debrecen, Hungary; szaloki.melinda@dental.unideb.hu; 2 Department of Experimental Physics, Institute of Physics, Faculty of Science and Technology, University of Debrecen, H-4026 Debrecen, Hungary; csarnovics.istvan@science.unideb.hu; 3 Department of Electronics Technology, Faculty of Electrical Engineering and Informatics, Budapest University of Technology and Economics, H-1521 Budapest, Hungary; bonyar@vik.bme.hu; 4 Wigner Research Centre for Physics, H-1121 Budapest, Hungary; 5 MTA-SZTE Lendület “Momentum” Noble Metal Nanostructures Research Group, University of Szeged, H-6720 Szeged, Hungary; ungord@chem.u-szeged.hu (D.U.); juhaszne.csapo.edit@med.u-szeged.hu (E.C.); 6 Interdisciplinary Excellence Center, Department of Physical Chemistry and Materials Science, University of Szeged, H-6720 Szeged, Hungary; 7 Department of Applied and Environmental Chemistry, University of Szeged, H-6720 Szeged, Hungary

**Keywords:** patchy silica, plasmonics, dental resin, green LED light, diametral tensile strength, differential scanning calorimetry, Raman spectroscopy, surface plasmon resonance imaging

## Abstract

A low ratio of polymerization is a major problem in resin-based composites. In this paper, the plasmonic effect of gold-covered silica nanoparticles on the physicochemical and mechanical properties of bisphenol A diglycidyl dimethacrylate (Bis-GMA), triethylene glycol dimethacrylate (TEGDMA) and urethane dimethacrylate (UDMA) green light-photopolymerizable dental resin was investigated at an intensity of 1.4 mW/cm^2^ for 40 s. Transmission electron microscopy (TEM) showed silica of about 350 nm covered with 12–15 nm gold nanoparticles (Au NPs) at 100% nominal coverage. Five different concentrations of bare and patchy silica particles were used; in the latter composite, the calculated Au wt% were 0.0052 wt%, 0.0104 wt%, 0.0208 wt%, 0.04160 wt%, and 0.0823 wt%. The plasmon peak of patchy silica-filled nanocomposite overlapped with the absorption of Irgacure 784 photoinitiator and green LED light emission peak. The effect of plasmon-enhanced polymerization achieved with green light illumination was analyzed using diametral tensile strength (DTS), differential scanning calorimetry (DSC), surface plasmon resonance imaging (SPRi), and degree of conversion (DC) based on Raman spectroscopy. The values of the Au NP with 0.0208 wt% was found to be maximum in all the measured data. Based on our result, it can be concluded that the application of patchy silica particles in dental resin can improve the polymerization ratio and the mechanical parameters of the composite.

## 1. Introduction

Resin-based dental composites are the most frequently used materials in dental practice. One of the advantages of light-curing resin-based composite is aesthetics; the filling process can be optimized, and handling of the filling materials is improved due to timed polymerization [1]. These composites have undergone continued developments since their introduction. These complex materials consist of a resin matrix, in which inorganic filler particles are embedded. The organic and inorganic phases are chemically bonded to each other through a silane coupling agent [1]. The organic resin matrix is a mixture of initiators, accelerators, and mono-, di- and trifunctional (meth)acrylate monomers. The acrylates form a cross-linked copolymer network through light-induced free radical polymerization. The latter process has several drawbacks, including polymerization shrinkage and stress, incomplete degree of polymerization, the presence and dissolution of unreacted monomers, and the inhibition effect of oxygen for polymerization [1]. The resin-based composite is strengthened by the manufacturers using inorganic fillers of different types, sizes, shapes, and ratios to provide enhanced mechanical and physical properties. The silane coupling agent tends to undergo hydrolysis in the humid oral environment, which weakens the bonding between fillers and the matrix. The shading effect of the filler limits the polymerization ratio. The blue light that activates the polymerization process loses its intensity after penetrating the material, and after a certain depth, it is no longer able to achieve a sufficient degree of polymerization efficiency (degree of double bond conversion). The propagation of light passing through a material is affected by many factors such as the filler particle size and quantity, and chemical components of the resin. The light is scattered when it reaches the boundary of a medium with a different refractive index. Significant light scattering develops on the surface of the small filler particles. At the same time, the resin continuously changes its refraction during polymerization (gelation and vitrification), and the refractive index continuously increases during polymerization [2]. Research revealed that light deflection, reflection, and refraction play a role in the question of polymerization depth to varying degrees. When the diameter of the particles reaches half the wavelength of the curing light, the waves interfere and cancel each other due to light bending. The filler particles, therefore, have a shadowing effect and can impair the effectiveness of the polymerization in the filler [3].

The above-mentioned drawbacks and consequences can lead to the deterioration of the clinical service life of the composite filling. Research on composites has taken a new direction, as the possibility of developing more properties according to the concept of functional fillers is being increasingly addressed at present. Six types of functions have been developed: improvement in physical properties, antibacterial effect, remineralization, self-healing property, a new type of X-ray shade solutions, and specific natural aesthetics similar to the tooth tissue [4]. Based on research, nano-sized metal particles like palladium are often used in nano metal catalysis for the creation of a C-C bond in the pharmaceutical industry [5,6,7]. The use of plasmons in dental composites can be considered as a new aspect of improvement in composites. The application possibilities of plasmonic nanoparticles and nanostructures are extensive in different fields, thanks to their special optical properties. The plasmon resonance is a wave-like pattern excited by an electromagnetic field of conduction electrons in a metal that can be classified into three groups according to its spatial extent: local, surface, and volume plasmon resonance. A metal nanoparticle excited by light of an appropriate wavelength emits part of its energy in the form of light into its environment, while another part absorbs and generates heat. The frequency of the emitted light can be influenced by the size and shape of the particle, and by changing the dielectric constant of the medium surrounding the particle. Both processes are closely related to the diameter of the particle, and the light-emitting and heat-generating capacity of the particle is distributed depending on the diameter. Spherical particles below 10 nm in diameter tend to generate heat, while large particles above 50 nm emit most of their energy in the form of light [8]. When multiple nanoparticles are used, the plasmons of the particles nearby interact with each other, and can influence each other’s effects by providing an enhancement effect [9]. The plasmonic heating effect of gold and silver nanoparticles was used to increase the degree of conversion (DC) of methacrylate resins during photopolymerization [10,11]. In our previous study, we used gold nanoparticles in dental resin [12]. The optimal gold concentration was 0.0208 wt% at 1.4 mW/cm^2^ of light intensity. 

In this study, gold-covered silica nanoparticles were applied in green light-photo-polymerizable dental resin, and their plasmonic effect on polymerization was also analyzed. It was assumed that better properties can be reached with at least the same or fewer gold particles than concentrations used in our previous study. The special arrangement of gold nanoparticles on the silica surface may eliminate the aggregation of plasmonic particles which can exert their effect more effectively. We expected that the degree of double bond conversion would increase, and thus, the mechanical and chemical properties will also improve due to the plasmon effect of patchy particles. During hand lamp polymerization of resin-based composites, a shadow effect may develop behind the silica particles because of the one-direction of light illumination. This effect can lead to a decrease in double bound conversion. With the application of patchy silica particles, the shadow effect of silica particles can be reduced during light illumination. 

## 2. Materials and Methods

### 2.1. Synthesis of Patchy SiO_2_ NPs

All designed nanoparticles were produced in our laboratory based on Dobrowolska et al. [13]. Silica particles were synthesized through a process based on the Stöber method. As a precursor, 8.5 mL tetraethoxysilane (TEOS, 98% reagent grade, Sigma–Aldrich Co., St. Louis, MO, USA) was dissolved in 100 mL ethanol (97%, VWR International LLC, Debrecen, Hungary). In a separate beaker, an ethanolic ammonia solution was prepared by mixing 35 mL ammonia solution (25 wt% concentration, VWR International LLC, Debrecen, Hungary) with 35 mL distilled water and 55 mL ethanol. The TEOS solution was added to the ethanolic ammonia solution dropwise at room temperature under constant stirring. The obtained white-colored dispersion was further stirred for 24 h. The precipitate was washed with a mixture of ethanol and distilled water and collected via centrifugation twice. The particles were dried at 80 °C overnight. For surface modification, 2 g silica was dispersed in 200 mL toluene (VWR International LLC, Debrecen, Hungary) and sonicated for 1 h. Then, 10 mL 3-aminopropyl-trimethoxysilane (APTMS, 97%, Sigma–Aldrich Co., St. Louis, MO, USA) was added. The mixture was stirred continuously and heated to 90 °C for 24 h under reflux. The silica particles were filtered and then washed twice via centrifugation in toluene at 13,000 rpm. The NH_2_-functionalized SiO_2_ NPs were dried at 80 °C overnight before gold deposition.

The gold nanoparticles (Au NPs) were prepared based on the well-known Turkevich method. Briefly, a 50 mL 5 mM HAuCl_4_ (99.9%, VWR International LLC, Debrecen, Hungary) solution was added into 18 mL ultrapure water. After reaching the boiling point, 50 mL 16.2 mM sodium citrate (98%, Molar Chemicals Ltd., Halásztelek, Hungary) solution was pipetted into the hot reaction mixture with continuous stirring until the permanent red color was obtained. The mixture was cooled to room temperature before further application. For the deposition of Au on the silica surface, 5 mg NH_2_-modified SiO_2_ NPs were dispersed into 10 mL ultrapure water via sonication for 1 h; then, it was mixed with 60 mL Au colloid dispersion for 4 h under vigorous stirring to reach the nominal 100% coverage. The composite particles were cleaned via centrifugation twice at 13,000 rpm. The final product was stored in powder form after freeze-drying. 

### 2.2. Synthesis of Patchy SiO_2_ NPs Containing Nanocomposite

The green light-photopolymerizable resin matrix composed of bisphenol A diglycidyl dimethacrylate (Bis-GMA; Sigma–Aldrich Co., St. Louis, MO, USA), triethylene glycol dimethacrylate (TEGDMA; Sigma–Aldrich Co., St. Louis, MO, USA) and urethane dimethacrylate (UDMA; Sigma–Aldrich Co., St. Louis, MO, USA) dimethacylate monomers was in a 21.4:25.4:53.3 weight ratio, respectively. The Irgacure 784 (Irg 784; BASF Hungary Ltd., Budapest, Hungary) green light-activated photoinitiator was applied in 5 wt% [12]. The composition of the resin matrix was constant; the patchy SiO_2_ particles were added to the resin mixture at five different concentrations. In repeated steps, sonication in an ultrasonic bath for five minutes, followed by a one-minute vortex, was applied to disperse the silica particles into the matrix. To investigate the plasmonic effect of gold on polymerization, five parallel controls (CTRL) were prepared for each sample containing the same size, type, and amount of silica particles without gold covering. The weight% (wt%) of patchy and bare silica particles and their abbreviated names are summarized in Table 1. 

### 2.3. Instruments for Patchy SiO_2_ Particles Characterization

Transmission electron microscopy (TEM) measurements were carried out with a Jeol JEM-1400plus (JEOL Ltd., Tokyo, Japan) instrument using 120 keV accelerating voltage. The samples were deposited on a carbon-covered copper grid. The size distribution was calculated with the ImageJ freely available Java software (https://imagej.en.softonic.com/download, accessed on 10 August 2023) based on 50–50 particles. The exact Au content of the patchy particles was measured with inductively coupled plasma mass spectrometry (ICP-MS) with an Agilent 7700X ICP-MS (Santa Clara, CA, USA) instrument equipped with an Agilent I-AS type autosampler. Before the analysis, Au was dissolved with hot aqua regia from the silica surface for 1 h, and the device was calibrated using the Agilent Multi-Element Calibration standard-3 for gold. 

### 2.4. UV-Vis Spectrometry Measurements of Nanocomposites

**Figure 1:**
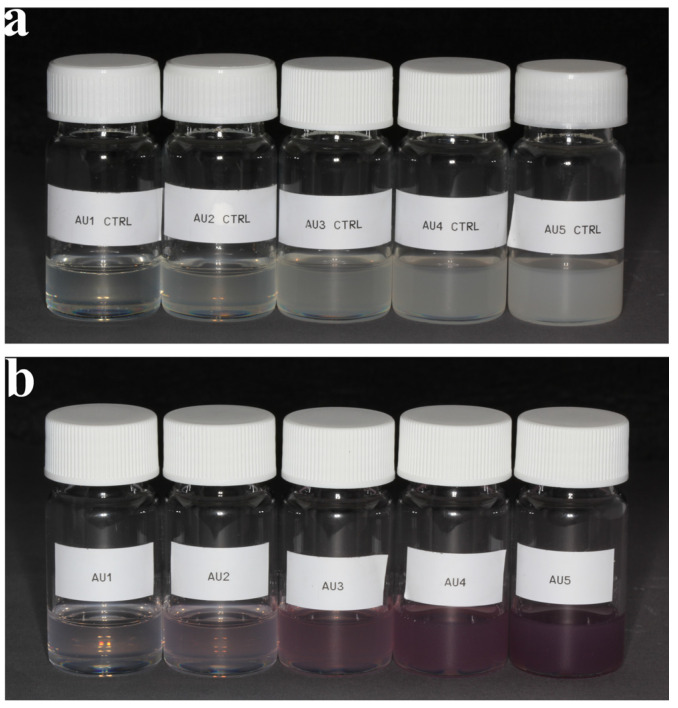
(**a**) Dental resin containing an increasing amount of bare silica nanoparticles without Irgacure 784 photoinitiator (Au1 CTRL, Au2 CTRL, Au3 CTRL, Au4 CTRL, and Au5 CTRL). (**b**) Dental resin containing an increasing amount of patchy nanoparticles without Irgacure 784 photoinitiator (Au1, Au2, Au3, Au4, and Au5).

The patchy and silica particles containing resin matrices without Irgacure 784 photoinitiator were used for UV-Vis spectroscopy measurements, which can be seen in Figure 1. Optical spectroscopy measurements of matrices were performed to obtain the localized surface plasmon resonance (LSPR) spectra. Optical spectroscopy measurements were performed with an Avantes Avaspec 2048-4DT spectrometer (Avantes, Apeldoorn, The Netherlands) and an Avantes Avalight DHS halogen light source. Small amounts of samples were dropped onto a glass slide, and thin layers (~500 µm) were measured in transmission mode. For each sample, the respective control (Figure 1, a part) was used as a reference, which was subtracted from the measured spectrum. This way the optical spectra only represent the effect of the gold nanoparticles. 

### 2.5. Specimen Preparation and Measurements of Diametral Tensile Strength (DTS)

The nanocomposite was placed into a Teflon mold, which was 3 mm in height and 6 mm in diameter. The surface of the sample was covered with a polyester foil to prevent the formation of an oxygen inhibition layer. The photopolymerization was performed with the help of a custom-made LED light source that was used and presented in our previous studies [12,14] at an intensity of 1.4 mW/cm^2^ for 40 s. The cured specimens were kept dry at room temperature for 24 h before mechanical testing. A universal mechanical analyzer (Instron 5544, Norwood, MA, USA) equipped with a 2 kN load cell was used for DTS measurements at 1 mm/min crosshead speed. The DTS data were calculated based on the following equation [12] (*n* = 10):

(1) $$DTS=\frac{2F}{\pi hd},$$

where F is the compression load (N), and h and d are the height and the diameter of the specimens, respectively. The *π* was a constant at 3.14.

### 2.6. Differential Scanning Calorimetry (DSC) Measurements of Nanocomposites

Perkin-Elmer DSC-7 Differential Scanning Calorimeter (Waltham, MA, USA) was used to determine the heat of post-polymerization (ΔH in J/g). The measurements were carried out in aluminum pans. An empty aluminum pan was used as a reference. Approximately, 35 mg samples were premeasured into the pans and were photopolymerized for 40 s at an intensity of 1.4 mW/cm^2^. After 24 h of standing (dry, room temperature, normal light condition), the samples were analyzed in a nitrogen atmosphere (1.0 cm^3^/s) at a heating rate of 10 °C/min in the temperature range 50–250 °C. The heat of the post-polymerization was calculated from the area under the peak of the differential temperature curve. The exothermic event peak temperature was determined by averaging three readings (*n* = 2).

### 2.7. Raman Spectroscopy Measurements of Nanocomposites

The degree of conversion (DC) of the created nanocomposites was studied with Raman Microscopy in different samples. The degree of conversion of the samples (*n* = 3) (parameters of the Teflon mold: 2 mm in height and 10 mm in diameter) was measured using Confocal Raman spectroscopy (Horiba LabRam HR Evo, Paris, France). The polymerization of tested nanocomposites was performed in a Teflon mold under a green LED light source for 40 s at an intensity of 1.4 mW/cm^2^. The polymerized specimens were kept dry at room temperature for 24 h before measurements. On the sample surface, 5 measurements were performed on different points. During the studies, a 633 nm laser is used to excite the samples for 10 s, and the accumulation is set to be 5. The excitation beam was focused onto the sample surface with 10× lens, while the 600 line/mm grating was used for the measurement. The intensity of the laser was reduced to <1 mW at the sample surface to avoid damage and light-induced transformation of the samples.

Spectra were baseline-corrected with the built-in algorithm of the Raman spectrometer software (https://www.renishaw.com/en/raman-software--9450, accessed on 10 August 2023), and then normalized and fitted with a set of Gaussians to obtain the Raman peak parameters. The error of the fitting and calculation of the investigated peaks, their parameters, and the estimation of the degree of conversion was about 0.5–1%. The degree of conversion was calculated based on the following equation:

(2) $$DC\left(\%\right)=100\times \left[\frac{\frac{{I2}_{polymerized}}{{I1}_{polymerized}}}{\frac{{I2}_{unpolymerized}}{{I1}_{unpolymerized}}}\right],$$

where I_1_ and I_2_ correspond to the area under the peaks at 1610 cm^−1^ and 1640 cm^−1^, respectively [15].

### 2.8. Surface Plasmon Resonance Imaging (SPRi) Measurements of Nanocomposites

The photopolymerization process was studied by measuring the change in the refractive index during irradiation caused by a green LED light source (output 1.4 mW/cm^2^), in a similar way as it was conducted in our previous paper [12]. The refractive index changes were measured with a custom-built Surface Plasmon Resonance imaging (SPRi) system described in Bonyar et al.’s research [16]. Real-time changes in the refractive index of the media were monitored during the whole duration of the illumination. The SPRi measurements were mainly designed for the analysis of aqueous solution; the instrument was recalibrated to higher refractive indexes substances such as the investigated nanocomposites. For kinetic measurements, SPRi chips (50 nm gold deposited on glass surface; Xantec, Germany) were used, on whose surface the investigated composites were dropped (*n* = 3). SPRi results (kinetics) provide information about the absolute changes in the refractive index of the nanocomposites with time. Due to the nature of SPRi measurements, only kinetics measured simultaneously on pairs of samples—on the same chip, at the same time—can be compared, which is true for all kinetics comparisons shown in our figures. A comparison between individual measurements (e.g., a comparison of kinetic curves from separate measurements) was not carried out.

### 2.9. Statistical Analysis

The statistical analysis for DC, DTS, and DSC data was performed using Student’s *t*-test with SPSS 17.0 software (IBM, Armonk, NY, USA). The significance level was set at 0.05. The data showed normal distribution, and variances were equal across groups based on the Kolmogorov–Smirnov Test and homoscedasticity Bartlett’s Test.

## 3. Results

### 3.1. Parameters of Patchy SiO_2_ Particles: Size and Gold Content

**Figure 2:**
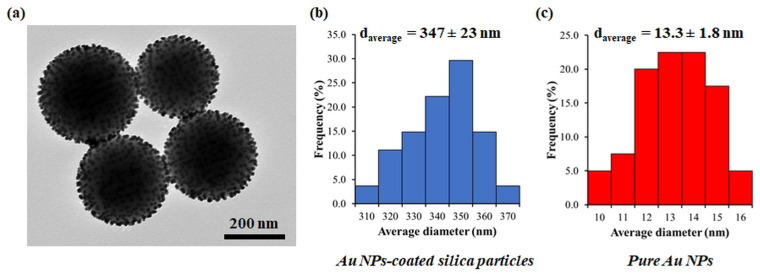
Representative TEM image of Au NPs-covered silica particles (**a**) with the size distribution diagram (**b**) and the size distribution diagram of pure Au NPs (**c**).

TEM characterization of patchy SiO_2_ particles revealed that the size of the prepared silica particles was about 350 nm. The diameter of gold nanoparticles was approx. 12–15 nm (Figure 2).

The ICP-MS measurements showed the exact gold content of the patchy particles, which was 0.3 mg Au/1 mg patchy silica. Based on this information, weight% of Au NPs in the resin matrix was calculated, and is summarized in Table 2. These data were necessary to compare the present study data with our previous works [12]. 

### 3.2. Results of UV-Vis Spectrometry Measurements

**Figure 3:**
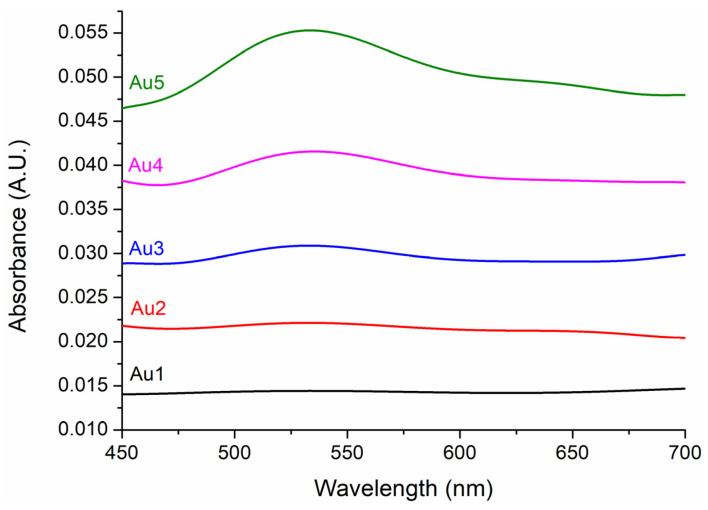
Offset optical absorption spectra of gold-covered silica nanoparticles-filled dental resin without Irgacure 784 photoinitiator with increasing filler concentrations (from Au1 to Au5).

The optical spectra of bare SiO_2_ and patchy SiO_2_ particle-filled resin matrices are summarized in Figure 3. 

The UV-Vis spectrometry measurements revealed an LSPR peak between 532 and 535 nm. The intensity of the LSPR peak increased with increasing gold concentrations. At low gold concentrations, namely, Au1 and Au2, the SPR peak was barely visible. 

### 3.3. Results of Diametral Tensile Strength Measurements (DTS)

**Figure 4:**
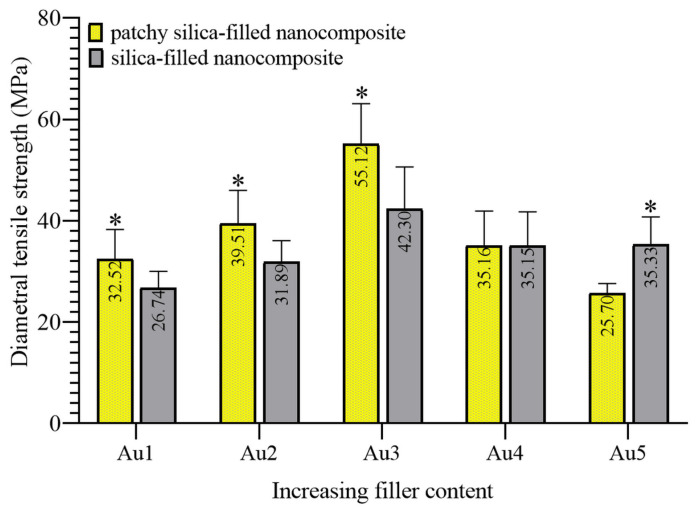
Mean diametral tensile strength data of patchy and bare silica-filled nanocomposites at five different filler concentrations (* significant differences compared to silica-filled, own control nanocomposite, *p* < 0.05).

DTS mechanical testing analyzes the cohesive force of cured composites. Polymerization causes volumetric changes similar to plasmon-enhanced polymerization. DTS is a suitable method for the analysis of this change. The calculated DTS data are summarized in Figure 4. 

The patchy SiO_2_-filled nanocomposite showed the highest strength with the Au3 composition. The DTS values of nanocomposites increased continuously till Au3 composition, and gold patchy silica-filled composites showed significantly higher strength than bare silica-filled nanocomposites, i.e., their controls. Au4 and its control composites showed similarities in DTS measurements; they were not significantly different. With the Au5 composition, the patchy particles-filled resin showed significantly lower DTS data than its control, i.e., the silica-filled nanocomposites. 

### 3.4. Results of Differential Scanning Calorimetry (DSC) Measurements

**Figure 5:**
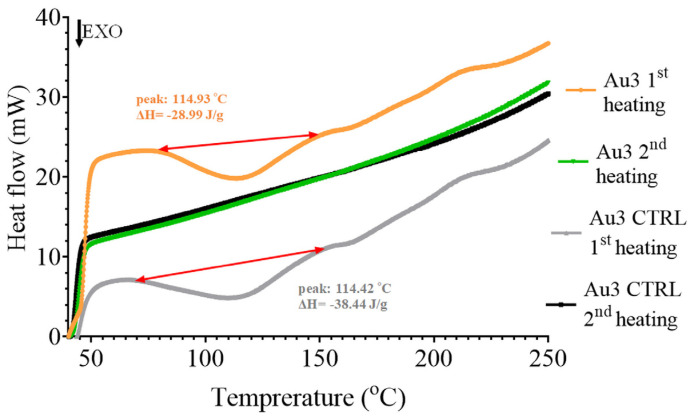
Representative differential temperature curves of first and second heating of Au3 patchy silica-filled and Au3 CTR bare silica-filled nanocomposites.

**Figure 6:**
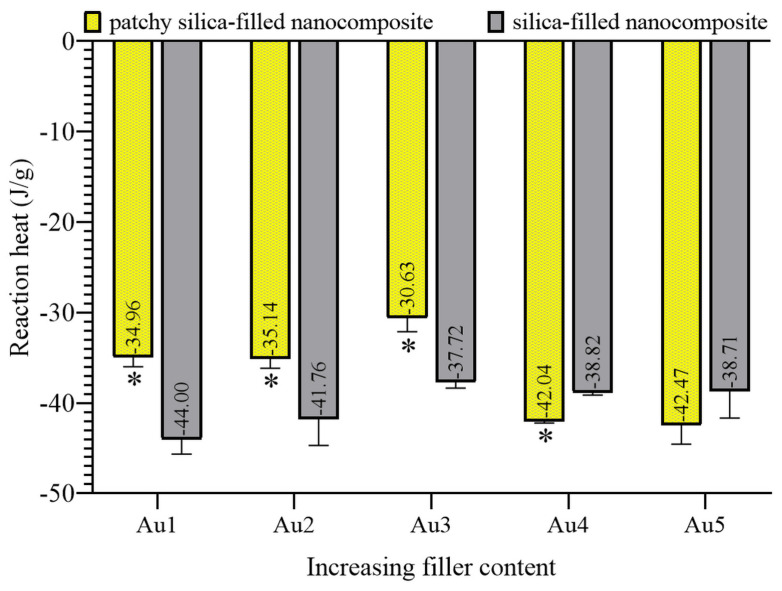
Mean exothermic reaction heat of patchy and silica-filled nanocomposites at five different filler concentrations (* significant differences compared to silica-filled, own control nanocomposite, *p* < 0.05).

DSC measurements were used to determine the post-polymerization reaction heat in Joule per gram, in which the pendant vinyl groups of polymer chains reacted to form a more cross-linked polymer network. This measurement provided indirect information about the degree of polymerization that is formed during the light illumination and dark phase of polymerization after 24 h of storage. Less reaction heat (closer to zero) meant a higher degree of polymerization in the cross-linked network formed during photopolymerization and a less amount of pendant vinyl groups. In the first heating cycle, the unreacted carbon double bonds reacted, resulting in an exothermic reaction heat. The peak of the exothermic temperature curves of nanocomposites was at around 114 °C. There is no peak at the temperature curve of second heating, justifying the complete crosslinking reaction of pendant vinyl groups, as visible in Figure 5. The mean values of the post-polymerization reaction heat of the first heating cycles are summarized in Figure 6. 

The calculated reaction heat decreased from Au1 to Au3, which meant that less unreacted vinyl groups remained after the green light-illuminated photopolymerization. The Au3 patchy silica-filled nanocomposite showed less reaction heat at −30.63 J/g. Till this filler concentration, the reaction heat of bare silica-filled composites was above patchy particles-filled nanocomposites, indicating the higher amount of pendant vinyl groups in partly cross-linked polymer networks. At higher filler concentrations, namely Au4 and Au5 patchy-filled composite, the reaction heats were higher than at Au3, and the control samples produced less reaction heat than the patchy particles-filled composites.

### 3.5. Results of Raman Spectroscopy Measurements

The degree of conversion of the created nanocomposites was analyzed to show the role of the concentration of gold nanoparticles in the photopolymerization process. The DC values were calculated from the Raman spectra, and recorded for the monomer and polymer states of the created nanocomposites. DC was different for the five samples and for the controls as well. The calculated DC data are summarized in Table 3. For all cases, the gold nanostructures-containing samples have a significantly larger value of conversion than the samples without them (*p* < 0.05). The DC value was maximum in the Au3 composite which contained 0.0208 wt% of Au (see Table 2). 

### 3.6. Results of Surface Plasmon Resonance Imaging (SPRi) Measurements

Refractive index change determined with SPRi measurements for silica and patchy silica-filled nanocomposites is shown in Table 4. The relative SPRi values are given, which also includes the signal change in Au3.

**Figure 7:**
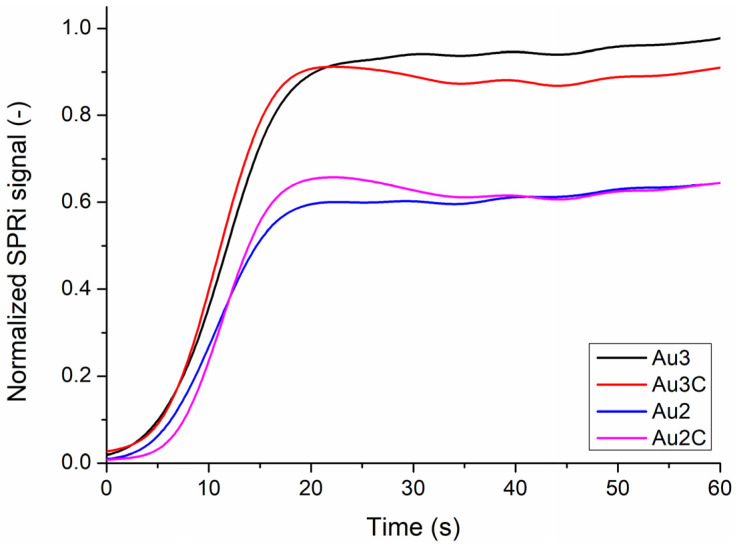
Representative noise-filtered SPRi curves: refractive index change during the photopolymerization of silica-filled control nanocomposites (Au2C and Au3C) and patchy silica-filled nanocomposites (Au2 and Au3).

The refractive index change in the patchy silica-filled nanocomposite increased from Au1 to Au3, reaching a maximum in Au3. In Au4 and Au5, the refractive index decreased compared to Au3. In the silica-filled nanocomposite, a similar tendency was observed, and the maximum change was observed in Au3 CTRL. The refractive index change value of the control and patchy particles-filled nanocomposites is very close to each other; there are no significant differences between them, except in Au3, where the difference was obvious and significant (*p* = 0.0013). For each sample, values in Au3 was significantly higher than the others. Au2 with Au4, and Au1 with Au5 showed similarities. In Figure 7, the representative SPRi kinetic curves are presented for Au2 and Au3 and their controls. The normalized SPRi signals reached the maximum value at around 40 s. In Au2 and its control, the signal showed similar values at 40 s. In Au3, the SPRi signal presented a higher value than its control at the end of light illumination. 

## 4. Discussion

In this study, gold-covered silica particles were used in five different concentrations in green light-photopolymerizable dental resin to increase the degree of polymerization of nanocomposites. The degree of conversion (DC) is never full in the resin-based composite. The unreactive monomers have a plasticizing effect, distorting the physicochemical and mechanical properties. DC depends on many factors including the chemical structure of monomers, initiation technique, curing time, sample thickness, applied initiator system, filler content, size, and size distribution [17]. The filler particles caused a shadow effect, which is well-known in a resin-based dental composite, that leads to a reduction in the degree of polymerization conversion. We aimed to increase DC in these shadow spaces using the plasmon-enhanced polymerization process. In our previous study, Au NPs without silica filler were used in dental resin. The aggregation of nanoparticles, due to their high surface energy, is a known problem in nanocomposite preparation [12,18]. Therefore, in this study, patchy or gold-covered silica particles were used to eliminate the agglomeration of Au NPs.

**Figure 8:**
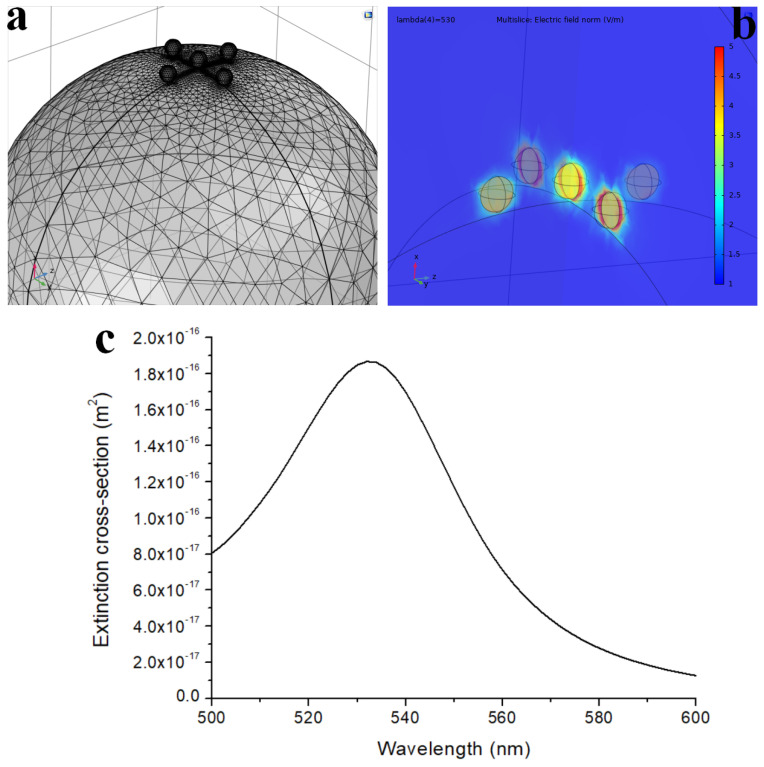
FEM model of the patchy nanoparticles. (**a**) The constructed 3D model and mesh. (**b**) The resulting electric field maps around the nanoparticles. (**c**) The resulting extinction spectrum with the LSPR peak at 532 nm.

TEM and ICP-MS analyses of patchy silica revealed that the surface of the silica spheres (diameter approx. 350 nm) was covered with Au NPs of 12–15 nm diameter, packed closely together. The 0.3 mg Au/1 mg patchy silica can be considered to have 100% coverage. The optical spectroscopy measurement is the most appropriate method for analyzing localized surface plasmon resonance of Au NPs. The plasmon peaks of patchy silica-filled nanocomposites were between 532 and 535 nm (as shown in Figure 3), overlapping with our green LED light emission peak and Irgacure 784 green light-activated photoinitiator absorption tail [12,14]. Therefore, the photopolymerization of dental monomers and plasmon-enhanced polymerization via green light illumination was provided simultaneously around 532 nm. To confirm that the observed peak indeed corresponds with the surface plasmon absorption of the nanocomposite, finite element modeling (FEM) was performed with Comsol 3.5. The diameter of the silica sphere was set to be 300 nm capped with 5 spherical gold nanoparticles with diameters of 12 nm. The particles’ position was set 10° apart from each other along the circumference of the silica sphere, as illustrated in Figure 8. The illumination was a plane wave propagating perpendicular to this arrangement with linear polarization. A perfectly matched layer (PML) with 700 nm diameter was also placed around the arrangement. The refractive index of the polymer matrix was set to be 1.53, based on our previous measurements with ellipsometry [19]. The resulting extinction spectrum has a definite LSPR peak around 532 nm (Figure 8c), which corresponds well with the experimentally measured data (see Figure 3). 

The plasmon-enhanced polymerization in patchy silica-filled nanocomposite and bare light-induced polymerization in silica-filled control composite showed differences in the degree of conversion based on Raman measurements. Patchy-filled samples had significantly larger values of conversion than the ones without them (*p* < 0.05) (Table 3). DC was maximum in Au3 which contained 0.0208 wt% of Au (Table 2). The DC data were around 71–77%, which can be considered as a higher value than the unfilled dental resin matrix [12]. The possible explanation for the increase in DC in patchy-filled nanocomposite was the plasmon effect upon polymerization. There was a correlation between the DC-determined Raman spectroscopy measurements and differential scanning calorimetry (DSC) measurements. The measured DC showed the polymerization degree of the crosslinked polymer network after 532 nm irradiation. Pre-photopolymerized samples were analyzed [19,20] with the DSC measurements, which provided information about post-polymerization of unreacted monomers, and pendant vinyl groups and chains. The exothermic event of the first heating cycle of pre-polymerized samples indicated post-polymerization, as a highly cross-linked network was formed. The polymer network defects like loops, entanglements, and physical crosslinks determined the crosslink density, and thus, the physicochemical and mechanical properties of the composite [17]. The cyclization reduced DC and the reaction heat of post-polymerization. Tanimoto et al. found that increasing the amount of filler caused no difference in the DSC peak, which aligns with our results [21]. 

**Figure 9:**
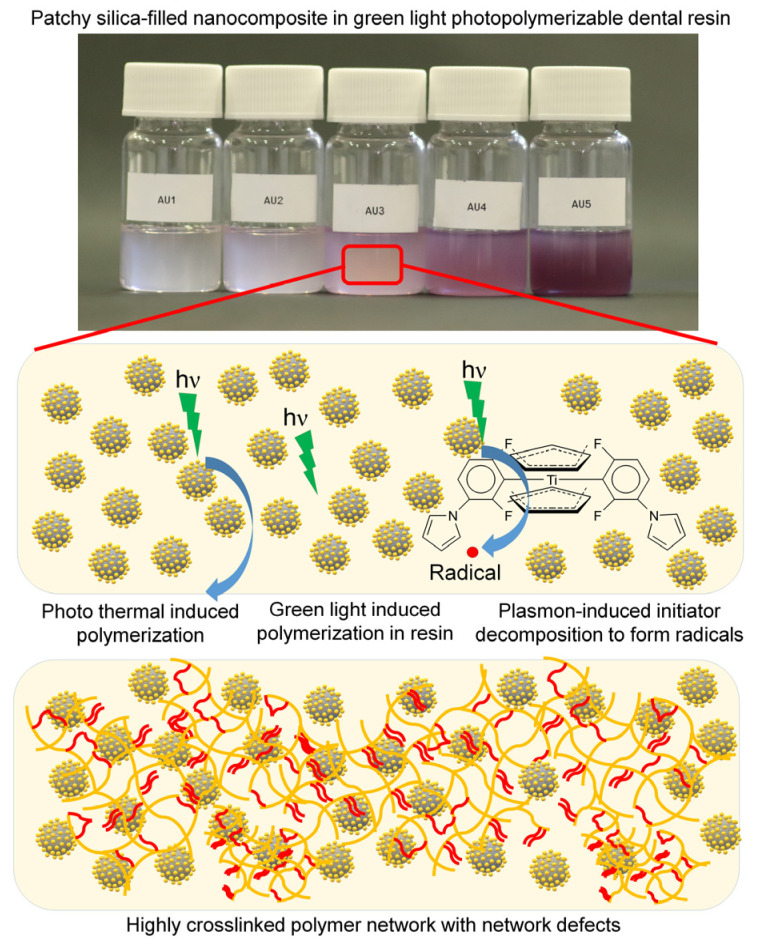
Schematic presentation of green light-induced photopolymerization, and the effect of patchy particles on photopolymerization.

DTS and DSC showed consonant results. The lower contents of gold concentration (Au1, Au2, and Au3) had a reinforcing effect on the cohesive strength of the composite, and showed less unreacted monomers and pendant vinyl groups compared to their control (silica-filled nanocomposite). During both measurements, the maximum value was observed in Au3 that corresponds to a concentration of 0.0208 wt% of Au. This concentration showed good agreement with our previous study [12]. The additional advantage of using patchy silica in dental resin was that the lower Au NPs concentrations (Au1 and Au2) were efficient for polymerization due to their structure. Au NPs were bonded chemically (through the ionic bond between the citrate covering of gold (negative) and the amino ending of silane (positive)) to the silica surface, ensuring the homogenous distribution of plasmons. The strengthening effect of plasmons in DC and DSC is due to the plasmon-enhanced polymerization effect near the gold nanoparticles. With the increase in plasmon concentration (from Au1 to Au3, by increasing the patchy silica filler amount), this effect multiplied. The size of the gold nanoparticles determined the absorbable light wavelength. The optimal matching of the size of Au NPs and the frequency of incident wavelength resulted in the collective motion of electrons and transient polarization of the particles to form surface plasmon resonance. The two main recommended mechanisms for energy dissipation were plasmon resonance energy transfer and plasmon-induced charge transfer, which inject hot electrons from metallic nanoparticles to the surrounding media [22,23,24]. These electrons may have a significant effect on free radical polymerization [25]. Plasmon relaxation may cause a few effects. One is that the relaxation can lead to a thermal effect. It can also lead to the antenna effect or induce NPs to take part in the electron transfer process (electron donor and electron acceptor) [26]. Finding out the relaxation route that is followed in a given system is a complex process and requires further experiments. Under 10 nm, the plasmon relaxation of spherical nanoparticles occurs via the thermal effect, and above 50 nm, it occurs mainly via light emission [8]. There are several studies in which plasmon-induced polymerization was investigated [10,19,25,26,27,28,29,30]. In most cases, the laser-induced plasmon effect was applied. In Asmussen et al., LED light-illuminated silver nanoparticles-generated plasmon effect was investigated for photopolymerizable methacrylate resin [10]. LED irradiation provided an inexpensive alternative to laser. The absorbed energy of plasmonic particles is converted quickly into heat that is transferred rapidly to the surrounding media, elevating the temperature of the medium much faster than in a vacuum [27]. This thermal-induced phenomenon worked at the nanoscale in a resin matrix, resulting in the formation of a thin layer of polymer around the plasmon nanoparticles from 10 nanometers up to 150 nanometers even without initiator [25], depending on the irradiation time and intensity [30]. The detailed chemical reaction in these few nano-sized volumes is unclear, and strongly depends on the chemical structure of polymerizable monomers. According to Wang and Ding et al., the hot electrons initiated the polymerization by producing radicals in monomer fractions close to the Au surface that bound to the metal surface and took part in propagation [25,30]. The formation of Au-C bonds was justified in previous reports [31,32]. Ding et al. also supposed the physical attachment between the polymer and plasmon particles [25]. The photon energy is less than 2 eV, which is insufficient to directly produce free radicals from monomers. In the above-mentioned studies, the resin does not contain initiators. Stamplecoskie et al. investigated plasmon-mediated photopolymerization in a mixture of trifunctional monomer, silver nanoparticles, and free radical initiator [28]. They described that the radicals were formed from the initiator due to plasmon-mediated excitation, and the radicals showed a transient binding to metal nanoparticles. It means that the triggering impact for polymerization occurred through the decomposition of the initiator via the plasmon effect. Similar results were found in another study. Au NPs may form a stable complex with Irgacure 784 photoinitiator [33,34] through donor–acceptor bonds between the aromatic rings (phenyl and/or pyrrole) of Irg 784 and positively charged gold nanoparticles as acceptors. If the Irg 784 is within the plasmon field of Au NPs under 532 nm irradiation, the destructive effect of Au NPs for the initiator exceeds the Au/Irg 784 complex stability. The outcome was that Au NPs facilitated the polymer chain formation around the metallic nanoparticles and increased the conversion (Figure 9). These nano-sized changes in conversion were reflected in the properties of bulk materials, as was visible in the Raman and DSC measurements. 

There can be three reasons behind this: plasmon field influence, injection of hot electrons, and local thermal effects [25,30,35]. It was found that the temperature increase depended on the plasmon concentration. At low silver nanoparticle contents (under 2500 ppm), there was a linear relationship between them [10]. At higher plasmon particle concentrations, the temperature profile was specified by a competition between light absorption and heat transference. Furthermore, the transmitted light decreased along the irradiation path owing to the absorption and scattering of incident light by plasmon particles. Thus, light absorption decreased due to sample thickness and plasmon heating of the nanoparticle. This disadvantageous effect was reflected in our mechanical (DTS) and thermal analysis (DSC) of nanocomposites with concentrations Au4 and Au5. Raman measurement is used to analyze a 1–2-µm thick layer of the polymer for DC calculation, and DSC is used to characterize the whole bulk [19]. 

The following conclusions were drawn regarding the presence of silica particles: Without Au NPs, the silica itself had a mechanical strengthening effect (Au1, Au2, and Au3) due to light scattering and reflection by particles that could be significant under 30 s curing time [18,36]. Generally, the maximum scattering of silica is achieved when its size is approximately half of the curing wavelength. Increased light scattering of micro-sized particles leads to lower DC. The nano-sized silica reduces the scattering, which increases DC [1]. At higher silica contents (Au4 and Au5), polymerization could be retarded due to the aggregation of nanoparticles and increased viscosity of resin that influences the mobility of macro radicals and macromolecules. Another reason could be the lack of similarity in refractive index between the resin and filler that caused the dispersion of light transmittance, resulting in insufficient DC and depth of cure [1,21]. With the application of silica particles in dental resin, the inhomogeneity of the network increased, and crack nucleation appeared at the resin-filler interface, influencing the integrity of materials against the stress. This may be the reason why the measured DTS values were smaller than in our previous measurement [12]. The resin matrix influences the strength of a given filler’s content, size, and geometry [37]. Researchers found that the polymerization rate increased with filler content under 5 wt% of silica. Above this, the polymerization rate decreased [18]. 

The SPRi monitors the refractive index change during the irradiation of the nanocomposite. Generally, the refractive index increases with polymerization [12,30]. The kinetic measurements demonstrated higher refractive indexes with Au NPs than bare silica, indicating polymerization enhancement. The highest refractive index change was at Au3 composition, which aligns with the DC, DTS, and DSC results. The saturation of the SPRi signal occurred earlier than in our previous study, where Au NPs were used in the same photopolymerizable dental resin but without a silica carrier [12]. 

The limitation of this study is the use of ultra-low filler content. Our further aim is to increase the inorganic filler content close to the wt% of a flowable dental composite with the application of bare and patchy silica in a mixed form. Furthermore, the advantageous effect of plasmon can be enhanced with increased light intensity, curing time, and using plasmon particles of different shapes (triangles and stars). 

## 5. Conclusions

The plasmon-enhanced polymerization for green light-photopolymerizable dental resin was implemented with the application of gold-covered silica particles in five different low-filler concentrations. The plasmon peak of Au NPs and absorption of Irgacure 784 photoinitiator are overlapped in the nanocomposites. It was shown that this structure could enhance DC, resulting in less post-polymerization heat in DSC compared to bare the silica-filled nanocomposite. Au NPs showed a maximum value in measured data at 0.0208 wt%. Below this concentration too, the reinforcing effect of Au NPs prevailed, but higher concentration has a reducing effect on measured data owing to the absorption and scattering of incident light by plasmon particles, thus decreasing the transmitted light in the polymerization layer thickness. These optical phenomena lead to a decrease in mechanical and physicochemical properties. Based on our results, it can be concluded that the patchy silica particles can enhance the ratio of polymerization, thereby having a favorable effect on the mechanical properties of their composites. In the future, the aim is to increase the silica filler content by mixing bare and patchy silica to the approximate filler level of a flowable dental composite. The additional advantage of using patchy particles in dental composites is reduced degradation of filling materials, increased service life, and efficient and economical application.

## Figures and Tables

**Table 1:** The composition of the investigated nanocomposites with abbreviated names.

	Patchy Silica-Filled Nanocomposite					Silica-Filled Nanocomposite				
	Au1	Au2	Au3	Au4	Au5	Au1 CTRL	Au2 CTRL	Au3 CTRL	Au4 CTRL	Au5 CTRL
filler particles (wt%)	0.0225	0.0451	0.0901	0.1801	0.3563	0.0225	0.0451	0.0901	0.1801	0.3563

**Table 2:** The weight% of filler particles and gold NPs in dental resin matrices.

	Patchy Silica-Filled Nanocomposite					Silica-Filled Nanocomposite				
	Au1	Au2	Au3	Au4	Au5	Au1 CTRL	Au2 CTRL	Au3 CTRL	Au4 CTRL	Au5 CTRL
filler particles (wt%)	0.0225	0.0451	0.0901	0.1801	0.3563	0.0225	0.0451	0.0901	0.1801	0.3563
Au NPs (wt%)	0.0052	0.0104	0.0208	0.0416	0.0823	0	0	0	0	0

**Table 3:** Mean degree of conversion (%) data of patchy silica and bare silica-filled nanocomposites at five different filler concentrations.

	Patchy Silica-Filled Nanocomposite					Silica-Filled Nanocomposite				
	Au1	Au2	Au3	Au4	Au5	Au1 CTRL	Au2 CTRL	Au3 CTRL	Au4 CTRL	Au5 CTRL
DC (%)	73.40 *	75.80 *	77.50 *	74.40 *	72.80 *	71.30	73.30	74.60	73.40	71.10
SD	0.70	0.70	0.80	0.70	0.60	0.50	0.60	0.50	0.60	0.50

* significant differences compared to silica-filled, own control nanocomposite (*p* < 0.05).

**Table 4:** Relative refractive index change in gold-patchy and silica-filled nanocomposites.

	Patchy Silica-Filled Nanocomposite					Silica-Filled Nanocomposite				
	Au1	Au2	Au3	Au4	Au5	Au1 CTRL	Au2 CTRL	Au3 CTRL	Au4 CTRL	Au5 CTRL
*n*	0.62	0.71	1.00	0.78	0.60	0.57	0.79	0.91	0.79	0.64
SD	0.093	0.022	0.013	0.070	0.042	0.095	0.090	0.047	0.107	0.027
